# Ultra-Processed Food Consumption and Mental Health and Sleep Quality in Turkish Adults: A Cross-Sectional Study

**DOI:** 10.3390/healthcare14050575

**Published:** 2026-02-25

**Authors:** Serap İncedal Irgat, Hande Bakırhan

**Affiliations:** 1 Department of Nutrition and Dietetics, Faculty of Health Sciences, Karamanoğlu Mehmetbey University, 70100 Karaman, Türkiye; 2 Department of Nutrition and Dietetics, Faculty of Health Sciences, Kahramanmaraş Istiklal University, 46060 Kahramanmaraş, Türkiye; handecekici@hotmail.com

**Keywords:** ultra-processed food, mental wellbeing, sleep quality, depression, anxiety, stress

## Abstract

**Background/Objectives**: Ultra-processed foods are thought to affect sleep and mental health. The aim of this study was to examine the relationships between ultra-processed food consumption and mental health (mental well-being, depression, anxiety, and stress) and sleep quality in Turkish adults. **Methods**: In this cross-sectional study (n = 2935), the status of adult individuals was determined via the Short Screening Questionnaire of Highly Processed Food Consumption (sQ-HPF), the Warwick-Edinburgh Mental Well-being Scale (WEMWBS), the Depression-Anxiety-Stress Scale 21 (DASS-21), the Beck Depression Inventory (BDI), and the Pittsburgh Sleep Quality Index (PSQI). Pearson correlation and multivariate path analysis were used to examine the relationships between the scales. **Results**: Forty-six percent of the participants had a minimal level of depression, and the vast majority of participants had poor sleep quality (84%). According to the sQ-HPF classification, individuals exhibiting high consumption had higher DASS-21, BDI, and PSQI scores but lower WEMWBS scores than individuals exhibiting low consumption (*p* < 0.001). The sQ-HPF was found to have independent and significant associations with mental well-being, depression, mood symptoms, and sleep quality. A 1-unit increase in the total sQ-HPF score led to a 0.26-unit decrease in the WEMWBS score (β = −0.05; *p* = 0.004). In the model established for depression (BDI), a 1-unit increase in sQ-HPF corresponded to a 0.32-unit increase in the BDI score (β = 0.09; *p* < 0.001). Similarly, sQ-HPF was found to be a positive and significant predictor of the total DASS-21 score (β = 0.11; *p* < 0.001). The sQ-HPF total score showed the strongest positive correlation with impaired sleep quality, with a 1-unit increase in sQ-HPF leading to a 0.13-unit increase in the PSQI score (β = 0.15; *p* < 0.001). **Conclusions**: The consumption of ultra-processed foods is associated with poorer mental health and sleep quality, regardless of age, sex, and socioeconomic factors.

## 1. Introduction

Mental disorders, which are increasingly prevalent worldwide, cause significant financial and social burdens on health systems and affect the well-being of individuals [1,2]. Depression and anxiety are the most frequently diagnosed mental health disorders, affecting one in ten people [3]. Furthermore, mental disorders are associated with an increased risk of chronic diseases, decreased quality of life, and worsening sleep problems, whereas sleep disorders can also lead to a further increase in psychological symptoms and deterioration in overall health [4,5]. Dietary habits, particularly the consumption of ultra-processed foods (UPFs), have emerged as modifiable risk factors [6]. Ultra-processed foods are industrially formulated products and typically contain additives such as colorants, flavor enhancers, added sugars, fats, salt, and preservatives [7]. Characterized by low nutritional value and high caloric density, UPFs are widely accessible, convenient (ready-to-eat or ready-to-heat), affordable, and palatable [8]. Several factors, including income growth, urbanization, changing workforce structures, demographic shifts in developed and developing countries, and increasing global time constraints, are leading to an increase in UPFs consumption [9]. An analysis of national food intake surveys from 36 countries indicated that ultra-processed foods contribute between 9% and 60% of total dietary energy intake [10]. Furthermore, evaluations of global UPF sales data and consumption patterns over the past decade reveal a growing trend toward an ultra-processed global diet, particularly in low- and middle-income countries, despite significant variation across countries and regions [10,11,12]. High consumption of UPFs has also raised public health concerns [13]. Cohort studies and meta-analyses have examined the health effects of UPFs; UPFs have been reported to be associated with obesity [14], type 2 diabetes [15], cardiovascular diseases [16,17], cancer [18,19], survival [20,21,22], mental health [12,23,24] and sleep [6,25,26].

UPFs have been shown to be associated with negative mental health outcomes such as sleep problems, anxiety, and depression [12]. Data suggest that individuals with high UPF consumption have a greater risk of depression, anxiety, and sleep problems [27]. Furthermore, one study indicated that higher UPF intake increased the likelihood of recurrent depressive symptoms [28].

There are numerous biological mechanisms that explain the link between UPFs and mental health. Possible harmful mechanisms include nutritional imbalances; overeating; decreased consumption of health-protective phytochemicals; and the consumption of toxic contaminants from processing or packaging, harmful additives and additive mixtures, resulting in inflammation, dysglycemia, dyslipidemia, microbiome dysbiosis, and renal or hepatic dysfunction [6,10,12].

Although studies have examined UPFs separately in relation to depression, anxiety, and sleep quality [27,28], there is no research that holistically evaluates UPFs in relation to mental well-being, depression, anxiety, and sleep quality. Owing to the lack of data in the literature and the inability to reach a clear conclusion, this issue needs to be investigated in depth. Accordingly, this study aims to evaluate the possible effects of UPFs on individuals’ mental health outcomes and sleep quality, taking into account sociodemographic characteristics, potential modifiers, and confounders.

## 2. Materials and Methods

### 2.1. Study Design and Sample Selection

Post hoc power analysis for sample size was performed using the G*Power 3.1.9.4 software package. In this study, a multiple regression analysis was planned to examine the effect of ultra processed food consumption on mental health and sleep quality in adults, and the statistical power of the sample size was evaluated based on this analysis. In power calculations based on regression analysis, the effect size must first be defined to ensure the study has sufficient statistical power. Accordingly, the reference effect size classifications proposed by Cohen (1988) were used in the calculations [29]. According to Cohen, the effect size (f^2^) value represents a small effect size of 0.02, a medium effect size of 0.15, and a large effect size of 0.35. For the analyses planned within the scope of this study, a small effect size (f^2^ = 0.02), a significance level of α = 0.05, and a sample size of n = 2935 were accepted. Based on these parameters, the calculation performed with the G*Power program determined that the statistical power of the study was 99.99%. This cross-sectional study was conducted between June and December 2025, with 2935 individuals identified through voluntary sampling. This study utilized a voluntary convenience sampling method, a type of non-probability sampling. Participants were reached through open calls made via online platforms and telephone. To increase sample diversity, participation of individuals from different socioeconomic backgrounds was encouraged; however, no random or probability-based method was used in sample selection. Individuals who agreed to participate were informed in writing about the purpose of the research and the implementation process, and their written informed consent was obtained. All data were collected face-to-face with the volunteer participants by the research team. The exclusion criteria included individuals under 18 and over 65 years of age, those diagnosed with depression or insomnia, those with psychological disorders, those taking medication for sleep problems, those following a special diet program, and pregnant or breastfeeding individuals.

A questionnaire was administered to participants who agreed to participate in the study. Data were collected on the basis of participant self-reports. The questionnaire consisted of a demographic information form (age, sex, marital and educational status, profession, and anthropometric measurements such as height and weight, etc.) and five scales [Short Screening Questionnaire of Highly Processed Food Consumption (sQ-HPF), Warwick-Edinburgh Mental Well-being Scale (WEMWBS), Depression-Anxiety-Stress Scale 21 (DASS-21), Beck Depression Inventory (BDI), and Pittsburgh Sleep Quality Index (PSQI)]. The Beck Depression Inventory (BDI) was used to assess the severity of depressive symptoms and to allow categorization according to established clinical severity thresholds. In addition, the Depression Anxiety Stress Scales-21 (DASS-21) was included to capture broader dimensions of emotional distress, encompassing depression, anxiety, and stress symptoms. The concurrent use of these instruments was intended to provide a more comprehensive assessment of affective symptomatology, with the BDI focusing on depressive severity and the DASS-21 assessing multidimensional emotional symptoms.

#### 2.1.1. Short Screening Questionnaire of Highly Processed Food Consumption (sQ-HPF)

In this study, ultra-processed foods are operationalized using the Short Screening Questionnaire of Highly Processed Food Consumption (sQ-HPF). The sQ-HPF, developed by Martinez-Perez et al. [30] and Erdoğan Gövez et al. conducted a study in Turkish to determine its validity and reliability in 2024 [31]. It was determined that the Cronbach’s alpha value was 0.65. The total score ranges from 0 to 11, with scores of 6 or above indicating a high level of ultra-processed food consumption [31].

#### 2.1.2. Warwick-Edinburgh Mental Well-Being Scale (WEMWBS)

To determine the mental well-being levels of individuals, the Warwick-Edinburgh Mental Well-being Scale, developed by Tennant et al. [32] and validated and proven reliable in Turkish by Keldal (Cronbach’s alpha coefficient 0.89), was used. The scale consists of 14 items and is scored on a 5-point Likert scale (1 = never to 5 = always). The lowest possible score is 14, whereas the highest is 70. A higher total score indicates a positive trend in an individual’s mental well-being [33].

#### 2.1.3. Depression Anxiety Stress Scale-21 (DASS-21)

The Turkish version of the Depression-Anxiety-Stress Scale Short Form (DASS-21), consisting of 21 items, was used to assess individuals’ levels of depression, anxiety, and stress. The validity and reliability of the Turkish version of the DASS-21 were established by Yılmaz et al. (2017) [34]. Cronbach’s alpha coefficient was determined to be 0.87 in the internal consistency calculations of the scale [34]. The DASS-21 is a 4-point Likert scale and includes 3 subdimensions (depression, anxiety, and stress). It is suitable for measuring depression, anxiety, and stress in both normal and clinical samples. Each factor consists of 7 items. There are no reverse items in the scale [34].

#### 2.1.4. Beck Depression Inventory (BDI)

The Beck Depression Inventory, which is used to determine the symptoms and severity of depression in individuals, has had its Turkish validity and reliability established by Hisli [35]. The scale measures the emotional, cognitive, and motivational symptoms of depression. A total score between 0 and 9 points is interpreted as “minimal depression,” a score between 10 and 16 points is interpreted as “mild depression,” a score between 17 and 29 points is interpreted as “moderate depression,” and a score between 30 and 63 points is interpreted as “severe depression.” [35,36].

#### 2.1.5. Pittsburgh Sleep Quality Index (PSQI)

The PSQI, developed by Buysse et al. [37] and validated and made reliable in Turkish by Ağargün et al., was used to evaluate the sleep quality of individuals. Seven components, each scored between 0 and 3, are summed to obtain a total PSQI score ranging from 0 to 21; higher scores indicate worse sleep quality. A total PSQI score between 0 and 4 is considered “good sleep quality,” whereas a score between 5 and 21 is considered “poor sleep quality” [38].

### 2.2. Statistical Analysis of Data

The data obtained from the research were analyzed via the IBM SPSS Statistics 31.0 and AMOS 31.0 software packages. The distribution characteristics of continuous variables are presented as the means ± standard deviations, whereas categorical variables are presented as numbers and percentages. The normality of the distribution of continuous variables was evaluated via skewness and kurtosis values. For the management of missing data, participants with a large amount of missing information were excluded from the analyses, while a partial imputation approach was applied for variables with only minimal missing data. Categorical variables were compared across groups using the chi-square (χ^2^) test. In cases involving more than two groups, Bonferroni correction was applied following a significant overall effect to account for the increased risk of Type I error due to multiple comparisons. Independent samples *t* tests and one-way ANOVA were applied for continuous variables. For cases where the overall test was significant, differences between groups were examined using post hoc tests. Specifically, for groups defined by BDI classification (minimal, mild, moderate, severe), multiple comparisons were conducted using the Tukey HSD post hoc test. In the tables, differences between groups indicated by different superscript letters (a, b, c, d) within the same row are considered statistically significant (*p* < 0.05). Pearson correlation analysis was used to evaluate the relationships between scales. The correlation coefficients were interpreted as low correlations between 0.00 and 0.30, moderate correlations between 0.30 and 0.70, and high correlations between 0.70 and 1.00. In the interpretation of the findings, emphasis was placed not solely on individual *p*-values but also on effect sizes and the overall patterns of the results. The predictive associations of ultra-processed food consumption (sQ-HPF) with mental well-being (WEMWBS), depression (BDI), mental symptoms (DASS-21), and sleep quality (PSQI) were examined using multivariable path analysis. Age, sex, marital status, education level, and socioeconomic status were included in the model as potential confounding variables. Results are reported using unstandardized path coefficients (B), standardized coefficients (β), *p*-values, and the proportion of variance explained (R^2^). In all analyses, the statistical significance level was accepted as *p* < 0.05.

## 3. Results

The general characteristics of the participants and their sociodemographic characteristics according to ultra-processed food consumption categories are given in Table 1. A total of 59.5% of the participants were female (n = 1745), 69.9% were married (n = 2051), and 54.2% were high school graduates. The average age of the participants was 28.4 ± 10.94 years, and the average body mass index was 24.5 ± 4.66 kg/m^2^. While no difference was found in gender distribution between ultra-processed food consumption categories (low or high), statistically significant differences were found in terms of marital status, education level, occupation, alcohol consumption, age, and body mass index (*p* < 0.001).

Table 2 compares participants’ ultra-processed food consumption, sleep quality, mental well-being and psychological symptoms. The mean total PSQI score of the participants was 8.3 ± 2.84, the mean sQ-HPF score was 5.4 ± 3.13, the mean WEMWBS score was 44.9 ± 15.59, and the mean BDI score was 13.5 ± 11.64. The mean scores for the DASS-21 subdimensions (depression, anxiety, and stress) were 6.1 ± 4.19, 6.4 ± 4.18, and 5.7 ± 4.23, respectively. A total of 46.1% of the participants had minimal depression, and the vast majority of participants had poor sleep quality (84%). Furthermore, individuals exhibiting high consumption according to the sQ-HPF classification had higher DASS-21, BDI, and PSQI scores but lower WEMWBS scores than individuals exhibiting low consumption (*p* < 0.001).

**Figure 1:**
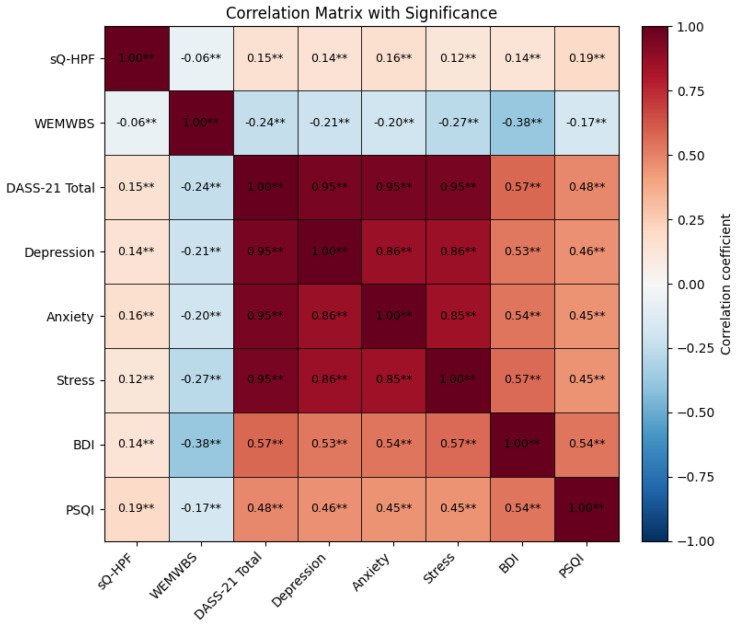
Pearson correlation matrix showing the relationships between ultra-processed food consumption, mental well-being indicators, and sleep quality. Positive correlations are shown in red, and negative correlations are shown in blue. The cell values represent Pearson’s r coefficients. BDI: Beck Depression Inventory; DASS-21: Depression, Anxiety and Stress Scale-21 Items; PSQI: Pittsburgh Sleep Quality Index; sQ-HPF: Screening Questionnaire of Highly Processed Food Consumption; WEMWBS: Warwick-Edinburgh Mental Wellbeing Scale. ** *p* < 0.01.

The results of the correlation analysis between ultra-processed food consumption (sQ-HPF) and mental symptoms, psychological well-being, and sleep quality are shown in Table 3 and Figure 1. Accordingly, the total sQ-HPF score was found to be positively and weakly correlated with the total DASS-21 score (r = 0.15; *p* < 0.001). Similarly, significant positive correlations were found between the sQ-HPF score and the BDI score (r = 0.13; *p* < 0.001) and the PSQI score (r = 0.19; *p* < 0.001). In contrast, a negative and weak correlation was found between sQ-HPF and the WEMWBS score, which indicates psychological well-being (r = −0.06; *p* < 0.001).

Comparisons based on depression and sleep quality classifications are shown in Table 4. As the severity of depression increased, the total PSQI, sQ-HPF, and DASS-21 scores increased significantly, whereas the WEMWBS score decreased significantly (*p* < 0.001). In the evaluation based on the PSQI classification, individuals with poor sleep quality had significantly higher total and subdimensional DASS-21 scores and BDI and sQ-HPF scores and lower WEMWBS scores than individuals with good sleep quality (*p* < 0.001).

**Figure 2:**
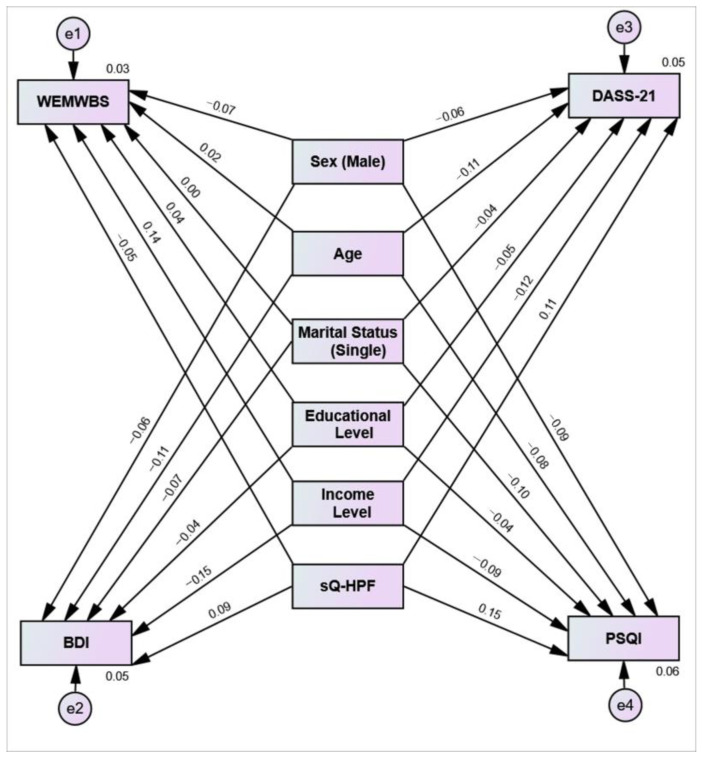
Multivariate path analysis model of the effects of ultra-processed food consumption on mental well-being, depression, psychological symptoms, and sleep quality.

In this study, age, sex, marital status, education level, and income level, which have been shown in the literature to be associated with both nutritional behaviors and mental health and sleep, were included in the multivariate path analysis model as potential confounding variables. The conceptual model of the established path analysis is presented in Figure 2, and the results of the multivariate analysis are shown in detail in Table 5. The consumption of ultra-processed foods was found to have independent and significant effects on psychological well-being, depression, mental symptoms, and sleep quality. In the model created for the WEMWBS, the total sQ-HPF score was found to be negatively correlated with psychological well-being, whereas a 1-unit increase in the total sQ-HPF score led to a 0.26-unit decrease in the WEMWBS score (β = −0.05; *p* = 0.004). In the model established for depression (BDI), a 1-unit increase in sQ-HPF corresponded to a 0.32-unit increase in the BDI score (β = 0.09; *p* < 0.001). Similarly, sQ-HPF was found to be a positive and significant predictor of the total DASS-21 score (β = 0.11; *p* < 0.001). In the sleep quality (PSQI) model, the total sQ-HPF score showed the strongest positive correlation with impaired sleep quality, with a 1-unit increase in the sQ-HPF leading to a 0.13-unit increase in the PSQI score (β = 0.15; *p* < 0.001).

Multivariate path analysis examining the effects of sQ-HPF, income, education, marital status, age and sex on BDI, PSQI, DASS-21 and WEMWBS scores. The model tested whether four dependent variables were significantly predictive of sQ-HPF. The dependent variables were the BDI, PSQI, DASS-21 and WEMWBS scores, while the independent variables were the total scores of the sQ-HPF, income, education, marital status, age and sex. Two-way arrows represent the correlation (r) between the scales, and the one-way arrows represent the standardized beta coefficients (β). The term “e” represents the margin of error, whereas the number in the dependent variable represents the explanatory variance of the independent variables in the dependent variable. Although these constructs are conceptually related, they were modeled as parallel outcome variables rather than as predictors of one another. This approach was adopted to avoid conceptual overlap within the model and to minimize potential concerns regarding multicollinearity.

## 4. Discussion

This study evaluated the effects of ultra-processed food consumption on mental health, psychological state, and sleep quality. Ultra-processed food consumption was found to be associated with decreased psychological well-being; increased symptoms such as depression, anxiety, and stress; and impaired sleep quality. These relationships persisted independently of age, sex, and socioeconomic factors. Our study did not find a statistically significant difference in UPF consumption based on sex. Although studies in the literature have shown that men consume more UPFs than women do [39,40], other studies have shown that women consume more UPFs [40,41]. Differences between genders are likely due to factors such as the level of development of the country, differences in the nutritional behavior of men and women, differing perceptions of healthy food among men compared with women, and the increasing attractiveness of ready-made meals due to the increasing number of working women [9,42]. The results of studies vary due to methodological, national, regional, and cultural differences. It is important to conduct longitudinal studies with large sample sizes, which are based on gender, at the national and international levels.

Factors such as age, gender, marital status, and employment status have also been found to be associated with UPF consumption [9]. Ge et al. [43] reported that married individuals consumed more UPFs, similar to our study. Furthermore, the study argues that married individuals have higher UPF consumption because they are among the vulnerable demographic groups [43]. Married individuals, owing to their busy work schedules and family responsibilities, tend to prefer quick and easy-to-prepare foods, which can increase their consumption of UPFs. Factors such as changing workforce structures, demographic shifts in developed and developing countries, and increasing global time constraints can also contribute to increased UPF consumption [9]. This issue can be clarified through studies examining various sociodemographic characteristics.

Our study revealed that the average age of individuals with low UPF consumption was greater than the average age of individuals with high UPF consumption. Similar results have been reported in studies in the literature, and UPF consumption decreases with age [44,45]. The consumption of these foods, characterized by high energy density and low nutritional quality, generally increases during late adolescence and young adulthood [46]. Consumption of ultra-processed foods generally declines with age, which may be explained by age-related changes in dietary preferences. Individual and environmental factors such as young individuals’ sensitivity to advertising, curiosity in trying new products, socialization needs, and insufficient knowledge and time to buy and cook healthy foods can influence these preferences [9,43,46]. Our study data revealed that UPF consumption is significantly associated with psychological symptoms, mental well-being, and sleep quality. A comprehensive review of UPFs and adverse health outcomes revealed that UPFs are associated with negative mental health outcomes such as sleep-related problems, anxiety, and newly emerged depression [12]. In our study, a 1-unit increase in the total sQ-HPF score corresponded to a 0.26-unit decrease in the WEMWBS score (β = −0.05; *p* = 0.004) and a 0.32-unit increase in the BDI score (β = 0.09; *p* < 0.001). Similarly, sQ-HPF was found to be a positive and significant predictor of the total DASS-21 score (β = 0.11; *p* < 0.001). In a cohort study (n = 4554) by Arshad et al. (2023), higher UPF intake was found to increase the likelihood of recurrent depressive symptoms by 34% [28]. A systematic review and meta-analysis revealed that higher UPF consumption was associated with an increased likelihood of common mental disorders (depressive and anxiety symptoms) [27]. Adjibade et al. reported that a 10% increase in the intake of ultra-processed foods was associated with a 21% increased risk of developing symptoms of depression over a 5-year follow-up period [47]. The relationship between UPF consumption and depression is thought to be linked to inflammatory processes and metabolic disorders. Ultra-processed foods are associated with chronic nutrition-related conditions, including obesity and metabolic syndrome, both of which exhibit bidirectional relationships with depression [48]. It is thought that such an outcome may occur due to the overactivity of the HPA axis, elevated cortisol secretion, immune-inflammatory signaling, leptin and insulin resistance, as well as potential alterations in the gut–brain [6,49]. Increased consumption of UPFs can lead to insufficient intake of healthy options containing important nutrients such as vitamins, minerals, and dietary fiber. Nutrient deficiencies can increase the risk of depression [6]. However, contaminants in food processing and phthalates found in packaging are reported to be linked to symptoms of depression [50]. Diets high in refined grains, simple sugars, and saturated or trans fats are associated with mental disorders, likely through pathways involving systemic inflammation, oxidative stress, and mitochondrial dysfunction [6,51]. Reduced dietary fiber intake can disrupt the balance of the gut microbiome via the gut–brain microbiome. It is also known that gut microbiome imbalances can further increase the risk of depression [6,52]. In conclusion, UPF consumption has the potential to negatively affect mental health through possible mechanisms of action; however, longitudinal and intervention-based studies are needed to reach a definitive conclusion. Since there is no research specifically examining this relationship in our country, it is important that future research examine this issue holistically with larger sample sizes and long-term follow-up.

Our study revealed that the total sQ-HPF score exhibited the strongest positive correlation with impaired sleep quality. A 1-unit increase in sQ-HPF was found to lead to a 0.13-unit increase in the PSQI score. There are also studies reporting a positive correlation between high UPF consumption and an increased risk of insomnia [27,53]. Pourmotabbed et al. (2024) reported a significant positive association between higher UPF intake and an increased risk of insomnia (OR = 1.53; 95% CI: 1.20, 1.95; I^2^ = 62.3%; *p* = 0.014; n = 7) [54]. Research using data from the NutriNet-Sante study reported that UPF consumption is associated with an increased likelihood of chronic insomnia [53]. It can disrupt sleep quality through multiple physiological pathways. These include increased levels of ghrelin and cortisol, which promote hunger and disrupt melatonin secretion; decreased leptin concentrations; disruption of the microbiota; and changes in serotonin and melatonin synthesis [26]. Furthermore, higher intake of added sugars and refined grains has been recognized as an independent risk factor for insomnia, leading to increases in the dietary glycemic index and glycemic load [55]. This is a very current topic, and more studies are needed to clarify it.

### Limitations

This study has several limitations. The fact that the sample consisted predominantly of young people and students (mean age: 28.4 ± 10.94; 46.1% students) limits the generalizability of the findings. Furthermore, because a non-probability convenience sampling method was used in the study, the results obtained do not statistically represent the entire adult population in Türkiye. Nevertheless, the findings contribute to understanding trends and patterns within the relevant sample group. Another limitation is that the data regarding the relationship between the severity of depression and sleep quality experienced by participants and UPFs are based on self-reports; this may lead to potential recall bias and misreporting. In this study, it is not possible to draw definitive conclusions because sleep quality, mental states, and nutritional characteristics are based on individuals’ self-assessments, perceptions, and memories. Data from individual reports may not reflect normal conditions or may lead to misinformation. Last limitation is that only sociodemographic covariates were included in the multivariate models, and that residual confounding from health behaviors and clinical variables is likely.

## 5. Conclusions

The study showed that consumption of ultra-processed foods is associated with poorer mental health and sleep quality, regardless of age, sex, and socioeconomic factors. Sleep quality was assessed solely via the PSQI, and more detailed clinical studies evaluating sleep quality could strengthen the research. To our knowledge, this topic has never been studied in a Turkish population before, and the uniqueness of this study is a strong point. This study is the first to holistically analyze the effects of UPFs on mental state, mental health, and sleep quality in a Turkish population, incorporating current nutritional concepts, which is another strong point of our study.

## Figures and Tables

**Table 1:** General characteristics of the participants.

Variables		Total (n = 2935)	sQ-HPF		*p*
			Low (n = 1486)	High (n = 1449)	
Sex n (%)	Female	1745 (59.5)	865 (58.2)	880 (60.7)	0.176
	Male	1190 (40.5)	621 (41.8)	569 (39.3)	
Marital status n (%)	Married	2051 (69.9)	917 (61.7) ^a^	1134 (78.3) ^b^	<0.001
	Single	884 (30.1)	569 (38.3) ^a^	315 (21.7) ^b^	
Education n (%)	Primary school	183 (6.2)	109 (7.3) ^a^	74 (5.1) ^b^	<0.001
	High school	1592 (54.2)	718 (48.3) ^a^	874 (60.3) ^b^	
	University	1056 (36.0)	599 (40.3) ^a^	457 (31.5) ^b^	
	Postgraduate	104 (3.5)	60 (4.0) ^a^	44 (3.0) ^a^	
Income status n (%)	Under minimum wage	252 (8.5)	114 (7.7) ^a^	137 (9.5) ^a^	0.038
	Minimum wage	1792 (60.1)	893 (60.1) ^a^	899 (62.0) ^a^	
	Above the minimum wage	892 (30.4)	479 (32.2) ^a^	413 (28.5) ^b^	
Occupation n (%)	Laborer	207 (7.1)	113 (7.6) ^a^	94 (6.5) ^a^	<0.001
	Officer	306 (10.4)	193 (13.0) ^a^	113 (7.8) ^b^	
	Employee in the business	422 (14.4)	211 (14.2) ^a^	211 (14.6) ^a^	
	Independent business	148 (5.0)	88 (5.9) ^a^	60 (4.1) ^b^	
	Retired	95 (3.2)	67 (4.5) ^a^	28 (1.9) ^b^	
	Student	1354 (46.1)	561 (37.8) ^a^	793 (54.7) ^b^	
	Unemployed	403 (13.7)	253 (17.0) ^a^	150 (10.4) ^b^	
Smoking n (%)	Yes	1021 (34.8)	477 (32.1) ^a^	544 (37.5) ^b^	0.002
	No	1914 (65.2)	1009 (67.9) ^a^	905 (62.5) ^b^	
Alcohol n (%)	Yes	530 (18.1)	203 (13.7) ^a^	327 (22.6) ^b^	<0.001
	No	2405 (81.9)	1283 (86.3) ^a^	1122 (77.4) ^b^	
Age (years, Mean ± SD)		28.44 ± 10.94	30.82 ± 12.07	25.99 ± 9.05	<0.001
Body mass index (kg/m^2^, Mean ± SD)		24.54 ± 4.66	24.83 ± 4.54	24.24 ± 4.77	<0.001

Note: The chi-square (χ^2^) test was used to compare categorical variables between groups, and the independent samples *t*-test was used for continuous variables. Values in the same row marked with different superscript letters (a, b) indicate statistically significant differences between groups after Bonferroni correction. sQ-HPF: Screening Questionnaire of Highly Processed Food Consumption.

**Table 2:** Participants’ ultra-processed food consumption, sleep quality, mental well-being and psychological symptoms.

Variables		Total (n = 2935)	sQ-HPF		*p* Value
			Low (n = 1486)	High (n = 1449)	
DASS-21 (Mean ± SD)	Total score	18.32 ± 11.97	16.56 ± 11.58	20.13 ± 12.1	<0.001
	Depression score	6.12 ± 4.19	5.54 ± 4.05	6.71 ± 4.24	<0.001
	Anxiety score	6.42 ± 4.18	5.77 ± 4	7.09 ± 4.26	<0.001
	Stress score	5.78 ± 4.23	5.25 ± 4.1	6.33 ± 4.28	<0.001
sQ-HPF Total Score (Mean ± SD)		5.48 ± 3.13	2.95 ± 1.78	8.08 ± 1.8	<0.001
WEMWBS Total Score (Mean ± SD)		44.91 ± 15.59	46.02 ± 15.51	43.77 ± 15.59	<0.001
BDI Total Score (Mean ± SD)		13.55 ± 11.64	12.08 ± 11.38	15.06 ± 11.71	<0.001
PSQI Score (Mean ± SD)		8.37 ± 2.84	7.85 ± 2.8	8.91 ± 2.77	<0.001
BDI classification n (%)	Minimal	1352 (46.1)	790 (53.2) ^a^	562 (38.8) ^b^	<0.001
	Mild	605 (20.6)	285 (19.2) ^a^	320 (22.1) ^a^	
	Moderate	651 (22.2)	274 (18.4) ^a^	377 (26.0) ^b^	
	Severe	327 (11.1)	137 (9.2) ^a^	190 (13.1) ^b^	
PSQI classification n (%)	Poor	2468 (84.1)	1172 (78.9) ^a^	1296 (89.4) ^b^	<0.001
	Good	467 (15.9)	314 (21.1) ^a^	153 (10.6) ^b^	

Note: The chi-square (χ^2^) test was used to compare categorical variables between groups, and the independent samples *t*-test was used for continuous variables. Values in the same row marked with different superscript letters (a, b) indicate statistically significant differences between groups after Bonferroni correction. sQ-HPF: Screening Questionnaire of Highly Processed Food Consumption; BDI: Beck Depression Inventory; DASS-21: Depression, Anxiety and Stress Scale-21 Items; PSQI: Pittsburgh Sleep Quality Index; WEMWBS: Warwick-Edinburgh Mental Wellbeing Scale.

**Table 3:** Correlation analysis between ultra-processed food consumption, psychological symptoms, mental well-being, and sleep quality.

Variable		sQ-HPF	WEMWBS	DASS-21	Depression	Anxiety	Stress	BDI	PSQI
sQ-HPF	r	1							
	*p*								
WEMWBS	r	−0.064 ***	1						
	*p*	<0.001							
DASS-21	r	0.150 ***	−0.240 ***	1					
	*p*	<0.001	<0.001						
DASS-21-Depression	r	0.144 ***	−0.215 ***	0.953 ***	1				
	*p*	<0.001	<0.001	<0.001					
DASS-21-Anxiety	r	0.162 ***	−0.199 ***	0.950 ***	0.861 ***	1			
	*p*	<0.001	<0.001	<0.001	<0.001				
DASS-21-Stress	r	0.122 ***	−0.270 ***	0.949 ***	0.857 ***	0.849 ***	1		
	*p*	<0.001	<0.001	<0.001	<0.001	<0.001			
BDI	r	0.135 ***	−0.378 ***	0.572 ***	0.528 ***	0.535 ***	0.567 ***	1	
	*p*	<0.001	<0.001	<0.001	<0.001	<0.001	<0.001		
PSQI	r	0.194 ***	−0.173 ***	0.477 ***	0.457 ***	0.449 ***	0.453 ***	0.538 ***	1
	*p*	<0.001	<0.001	<0.001	<0.001	<0.001	<0.001	<0.001	

*** *p* < 0.001, r: Pearson correlation coefficient. BDI: Beck Depression Inventory; DASS-21: Depression, Anxiety and Stress Scale-21 Items; PSQI: Pittsburgh Sleep Quality Index; sQ-HPF: Screening Questionnaire of Highly Processed Food Consumption; WEMWBS: Warwick-Edinburgh Mental Wellbeing Scale.

**Table 4:** Comparison of psychological symptoms, ultra-processed food consumption and mental well-being scores by depression level and sleep quality categories.

		BDI Classification					PSQI Classification		
		Minimal (n = 1352)	Mild (n = 605)	Moderate (n = 651)	Severe (n = 327)	*p*-Value	Poor (n = 2468)	Good (n = 467)	*p*-Value
DASS-21	Total Score	11.91 ± 9.39 ^a^	19.11 ± 8.12 ^b^	24.68 ± 10.55 ^c^	30.72 ± 13.16 ^d^	<0.001	19.79 ± 11.72	10.57 ± 10.2	<0.001
	Depression	4.04 ± 3.36 ^a^	6.48 ± 3.09 ^b^	8.13 ± 3.87 ^c^	10.09 ± 4.60 ^d^	<0.001	6.62 ± 4.11	3.51 ± 3.56	<0.001
	Anxiety	4.28 ± 3.35 ^a^	6.77 ± 3.01 ^b^	8.61 ± 3.76 ^c^	10.28 ± 4.69 ^d^	<0.001	6.92 ± 4.09	3.77 ± 3.59	<0.001
	Stress	3.60± 3.23 ^a^	5.86 ± 3.00 ^b^	7.95 ± 3.84 ^c^	10.35 ± 4.61 ^d^	<0.001	6.25 ± 4.18	3.29 ± 3.52	<0.001
sQ-HPF score		4.96 ± 3.21 ^a^	5.75 ± 2.83 ^b^	6.06 ± 2.96 ^b^	6.00 ± 3.30 ^b^	<0.001	5.67 ± 3.08	4.51 ± 3.19	<0.001
WEMWBS score		50.02 ± 15.58 ^a^	46.52 ± 12.66 ^b^	38.58 ± 13.63 ^c^	33.4 ± 13.68 ^d^	<0.001	44.35 ± 15.18	47.86 ± 17.32	<0.001
BDI score		3.98 ± 2.77 ^a^	12.8 ± 1.97 ^b^	22.21 ± 3.62 ^c^	37.29 ± 7.58 ^d^	<0.001	14.96 ± 11.76	6.13 ± 7.39	<0.001
PSQI score		6.93 ± 2.22 ^a^	8.62 ± 2.40 ^b^	9.73 ± 2.60 ^c^	11.17 ± 2.74 ^d^	<0.001	9.12 ± 2.45	4.44 ± 0.69	<0.001

Independent samples *t* tests were used for variables with two groups, and one-way analysis of variance (one-way ANOVA) was used for variables with three groups or more. For variables showing a statistically significant overall effect, multiple comparisons were performed using the Tukey HSD post hoc test. Groups denoted by different superscript letters (a, b, c, d) within the same row are considered to differ significantly (*p* < 0.05). BDI: Beck Depression Inventory; DASS-21: Depression, Anxiety and Stress Scale-21 Items; PSQI: Pittsburgh Sleep Quality Index; sQ-HPF: Screening Questionnaire of Highly Processed Food Consumption; WEMWBS: Warwick-Edinburgh Mental Wellbeing Scale.

**Table 5:** Multivariate path analysis results of the effects of ultra-processed food consumption on mental well-being, depression, psychological symptoms, and sleep quality.

Dependent Variables	Path	Independent Variables	B	S.E.	β (Beta)	*p*	
WEMWBS	<---	Gender (male)	−2.16	0.58	−0.07	<0.001	0.028
WEMWBS	<---	Age	0.02	0.03	0.02	0.398	
WEMWBS	<---	Marital status (single)	0.08	0.62	0.00	0.904	
WEMWBS	<---	Educational level	1.04	0.43	0.04	0.016	
WEMWBS	<---	Economic status	3.66	0.49	0.14	<0.001	
WEMWBS	<---	sQ-HPF	−0.26	0.09	−0.05	0.004	
BDI	<---	Gender (male)	−1.30	0.42	−0.06	0.002	0.049
BDI	<---	Age	−0.11	0.02	−0.11	<0.001	
BDI	<---	Marital status (single)	−1.69	0.45	−0.07	<0.001	
BDI	<---	Educational level	−0.63	0.32	−0.04	0.047	
BDI	<---	Economic status	−2.89	0.35	−0.15	<0.001	
BDI	<---	sQ-HPF	0.32	0.07	0.09	<0.001	
DASS-21	<---	Gender (male)	−1.51	0.43	−0.06	<0.001	0.045
DASS-21	<---	Age	−0.12	0.02	−0.11	<0.001	
DASS-21	<---	Marital status (single)	−1.14	0.47	−0.04	0.014	
DASS-21	<---	Educational level	−0.82	0.33	−0.05	0.012	
DASS-21	<---	Economic status	−2.36	0.37	−0.12	<0.001	
DASS-21	<---	sQ-HPF	0.40	0.07	0.11	<0.001	
PSQI	<---	Gender (male)	−0.50	0.10	−0.09	<0.001	0.056
PSQI	<---	Age	−0.02	0.01	−0.08	<0.001	
PSQI	<---	Marital status (single)	−0.63	0.11	−0.10	<0.001	
PSQI	<---	Educational level	−0.16	0.08	−0.04	0.041	
PSQI	<---	Economic status	−0.43	0.09	−0.09	<0.001	
PSQI	<---	sQ-HPF	0.13	0.02	0.15	<0.001	

Note: A multivariate path analysis was performed. B = unstandardized path coefficient; β = standardized path coefficient. Age, sex, marital status, educational level, and economic status were included as covariates. Statistical significance: *p* < 0.05, *p* < 0.01. R^2^ represents the variance explained in the dependent variables. BDI: Beck Depression Inventory; DASS-21: Depression, Anxiety and Stress Scale-21 Items; PSQI: Pittsburgh Sleep Quality Index; sQ-HPF: Screening Questionnaire of Highly Processed Food Consumption; WEMWBS: Warwick-Edinburgh Mental Wellbeing Scale.

## Data Availability

The datasets generated and/or analyzed during the current study are not publicly available due to restrictions (e.g., they contain information that could compromise the privacy of research participants) but are available from the corresponding author (Serap Incedal Irgat, email: serapincedal@kmu.edu.tr) upon reasonable request.

## Abbreviations

The following abbreviations are used in this manuscript:

sQ-HPF	Short Screening Questionnaire of Highly Processed Food Consumption
WEMWBS	Warwick-Edinburgh Mental Well-being Scale
DASS-21	Depression-Anxiety-Stress Scale 21
BDI	Beck Depression Inventory
PSQI	Pittsburgh Sleep Quality Index
UPFs	Ultra-processed foods
